# Effect of instrumented spinal fixation on outcome in polytrauma patients in the ICU

**DOI:** 10.1186/cc11063

**Published:** 2012-03-20

**Authors:** G Simpson, C Menakaya, A Bidwai, R Pillay, M Dematas

**Affiliations:** 1 Royal Liverpool University Hospital, Liverpool, UK

## Introduction

Spinal injuries in polytrauma patients carry high morbidity and mortality often necessitating intensive care admission. A review of polytrauma patients admitted to the ICU at The Royal Liverpool University Hospital was undertaken to investigate the effect of spinal instrumentation on outcome in the ITU.

## Methods

A retrospective review of all polytraumatized patients admitted to the RLUH ICU over 3 years with a thoraco-lumbar spinal fracture. Clinical records, laboratory results and radiological records were accessed. Patients were grouped according to the use of instrumented spinal fixation versus conservative management and outcomes compared.

## Results

Fourteen polytrauma patients with spinal fractures were admitted to the ICU over 3 years, five managed conservatively with a TLSO brace and nine managed operatively with instrumented spinal fixation. The degree of injury as graded by the Injury Severity Scale (ISS) was lower in the nonoperative group (mean: 27, range: 14 to 59) compared to the operative group (mean: 36.1, range: 14 to 57). Mortality was significantly higher in patients conservatively managed (nonoperative: 60%, operative: 0%) (*P *< 0.01). The intubation time was lower in patients who underwent spinal instrumentation (mean: 12.3 days, range: 1 to 27 days), when compared to conservative management (mean: 16 days, range: 11 to 27 days), and similarly the ITU length of stay was reduced in the operative group (operative: mean 20.6 days, nonoperative: 32.25 days). Development of respiratory failure was decreased in patients treated with instrumented fixation (operative 33.3%, nonoperative: 71%).

## Conclusion

Surgical stabilization of spinal fractures avoids restrictive spinal braces and permits mobilization. Surgical fixation of spinal fractures appears to decrease mortality and ITU stay and has a beneficial effect on respiratory function, with regards to degree of ventilatory support and development of respiratory failure.

